# Occult Untreated Alcohol Use Disorder in a Patient with Recurrent Pituitary Macroadenoma

**DOI:** 10.1155/2022/6825941

**Published:** 2022-01-06

**Authors:** Mckenzie P. Rowe, Alëna A. Balasanova

**Affiliations:** ^1^College of Medicine at the University of Nebraska Medical Center, Omaha, NE, USA; ^2^Department of Psychiatry, University of Nebraska Medical Center, Omaha, NE, USA

## Abstract

Alcohol use disorder (AUD) is a chronic relapsing and remitting psychiatric condition associated with adverse health outcomes. Although common, AUD is underdiagnosed, and treatment is often overlooked. At times of increased risk, such as the postoperative period, it is imperative to screen for and treat AUD to improve patient outcomes. Psychiatrists can play an important role in addressing AUD in this patient population through addiction psychiatry consultation services. We present the case of a patient with occult alcohol use disorder (AUD) leading to hospitalization in the setting of depressive mood symptoms and personality changes after a repeat pituitary macroadenoma resection and radiation five months earlier. AUD was noted months prior to hospitalization but was not addressed despite regular interactions with the healthcare system. Evaluation by addiction psychiatry specialists during hospitalization prompted recognition and treatment of AUD, resulting in cessation of alcohol use and resolution of mood symptoms and personality changes. The patient was discharged 3 days after admission and maintained abstinence from alcohol at two months postdischarge without recurrence of psychiatric symptoms.

## 1. Introduction

Alcohol use disorder is a common and chronic medical condition with an estimated prevalence of 7-25% among medically and surgically hospitalized patients [1]. Despite the high prevalence of AUD among inpatients, this diagnosis often goes undetected and therefore unaddressed [2, 3]. Of cases of AUD which are identified, only half have clinical documentation of any intervention or treatment referral for alcohol use [3]. Interaction with the healthcare system, particularly during hospitalization, presents an opportune time for evaluating and intervening on a patient's alcohol use. One study found that 43% of individuals with AUD who are inpatient, compared with 16% of individuals with AUD in the general outpatient population, were at a stage of change in which they were ready to engage in behavior change [3]. One useful intervention in the hospital setting is the use of psychiatric consultation-liaison services, including addiction consult services. Addiction consult services have been shown to significantly reduce addiction severity among those who use alcohol and other substances, improve treatment retention, increase the number of days of abstinence, and reduce alcohol consumption at 6 months postintervention [4].

In this case report, we present a patient with occult AUD in the setting of pituitary macroadenoma treatment, which was not addressed despite regular interaction with the healthcare system. Evaluation by addiction psychiatry specialists during hospitalization prompted treatment of AUD, resulting in cessation of alcohol use and resolution of psychiatric symptoms. The patient remained free from alcohol at least two months later without recurrence of psychiatric symptoms.

## 2. Case Presentation

A 36-year-old Black woman was brought to the emergency department for agitation and alcohol intoxication. Family had called 911 due to what they described as a “nervous breakdown” by the patient. Additionally, they reported agitation in the preceding weeks, such as “meanness” and irritability not characteristic of the patient's baseline demeanor. Pertinent findings on arrival included a blood alcohol level of 255 and urine drug screen positive for cannabinoids. The patient was admitted for medical management of alcohol withdrawal.

The patient's past medical history included AUD, nonfunctional pituitary macroadenoma, adrenal insufficiency, and hypothyroidism. The patient was status post-transsphenoidal pituitary tumor resection surgery in 2014 with multiple repeat resections, the most recent of which (her fourth resection) was 5 months prior to hospitalization and followed by a course of postoperative radiation therapy. The patient's medical record revealed “heavy alcohol use” was noted two years previously but was not followed. “Alcohol Abuse” was added to the patient's problem list six months prior to admission though no counseling or recommendations were made, nor was this addressed in subsequent follow-up appointments with the various healthcare providers she encountered spanning from primary care to a variety of surgical and medical specialists.

During hospitalization, the patient was initiated on the Clinical Institute Withdrawal Assessment Alcohol Scale with oral lorazepam. The addiction psychiatry team was also consulted to evaluate the patient's alcohol use and psychiatric symptoms. On interview, the patient reported a long history of social alcohol use but noted that her intake had sharply increased and became “a problem” 5 months prior, immediately following her most recent operation. She described her mood as depressed and anxious during those months due to ongoing postoperative medical needs that required her to take time off work, contributing to social isolation. Since that time, the patient reported drinking 1 pint of vodka on most days of the week and reported distress and guilt about her drinking. The patient's reported distress and impairment from alcohol use met the Diagnostic and Statistical Manual of Mental Disorders, Fifth Edition criteria for moderate alcohol use disorder, and her mood and personality changes preceding admission were thought to be substance-induced from the central nervous system effects of acute alcohol intoxication superimposed on chronic excessive alcohol use. The differential diagnoses considered also included abnormal hormone levels status postpartial pituitary resection, medication nonadherence for hormone replacement, adjustment disorder, major depressive disorder, and substance-induced depressive disorder. The patient did not demonstrate agitation or aggressive behaviors during her hospitalization, and per collateral sources, the personality changes she had exhibited at home had resolved with cessation of alcohol use and treatment of withdrawal.

The patient expressed a desire for sobriety and interest in pursuing multimodal treatment. She requested information about community mutual-aid groups and help setting up individual psychotherapy. The patient was amenable to initiating pharmacotherapy for AUD with extended-release naltrexone and received her first intramuscular injection prior to discharge from the hospital. The patient voiced optimism about her treatment plan and was discharged on hospital day 3 after an uncomplicated withdrawal course. One month later at her primary care visit, the patient reported ongoing abstinence from alcohol and requested a second injection of extended-release naltrexone. Two months later, the patient endorsed continued sobriety. The patient did not report any depressive mood symptoms at that visit, and the previously noted personality changes had not recurred.

## 3. Discussion

Endocrinologic abnormalities, including those of the pituitary, are known to be associated with psychiatric symptoms [5]. When comparing treatments of pituitary adenomas, quality-of-life measures in patients who receive radiation therapy show greater depression severity and deficits in social relationships compared to those of patients who do not undergo radiation [6]. Studies also show that anxiety, depression, substance use, and other psychiatric symptoms may flare in the postoperative period [7]. Depression and anxiety are additionally associated with alcohol addiction and are particularly prominent during the acute withdrawal phase [8].

Accordingly, the patient's postoperative and postradiation status may have contributed to the mood symptoms and personality changes exhibited by the patient prior to admission. However, addiction psychiatry consultation revealed that it was most likely excessive alcohol use and development of an alcohol use disorder that were the predominant sources of the patient's psychiatric symptoms, particularly owing to their resolution with recognition and treatment of AUD. Excessive alcohol use is more common among surgical patients and is associated with more postoperative complications and reduced immune capacity [7, 9–11]. Therefore, monitoring for the presence and severity of postoperative alcohol use is pertinent to patient outcomes.

The patient had many encounters with the healthcare system in the months following her fourth resection, resulting in missed opportunities to address her alcohol use. One way to address substance use in postoperative patients is during the “intervenable moment” of hospitalization, when an addiction psychiatry consult service can be utilized as an important inpatient intervention. Psychiatrists have the training and skills to comprehensively evaluate a patient's psychiatric symptoms in context of substance use and other medical conditions. After the addiction psychiatry team informed the patient of her treatment options, she became interested in and initiated multimodal treatment which led to resolution of her psychiatric symptoms.

The clinical significance of this case is that undiagnosed and untreated AUD can result in mood symptoms that can go unrecognized or be misattributed to other sources; with prompt recognition of and treatment of AUD, such mood symptoms may resolve. This case further augments the literature supporting evaluation for AUD in the postoperative period. This case also uniquely demonstrates the utility of addiction psychiatry consultation in addressing alcohol use in this patient population. In this case, cessation of alcohol use through medically managed withdrawal and initiation of pharmacotherapy for AUD with extended-release naltrexone during hospitalization resulted in resolution of the patient's depressive mood symptoms and personality changes.

## Data Availability

The authors confirm that all the information regarding the patient can be found within the article.
